# Effectiveness of a Transtheoretical Model-Based Intervention to Improve Blood Pressure Control of Hypertensive Patients in China: A Clustered Randomized Controlled Trial

**DOI:** 10.3389/fpubh.2021.760421

**Published:** 2022-01-25

**Authors:** Ping Chen, Ying Shen, Chao He, Xinying Sun

**Affiliations:** ^1^School of Public Health, Peking University, Beijing, China; ^2^Global Health Office, Beijing Center for Disease Prevention and Control, Beijing, China; ^3^Shunyi Center for Disease Prevention and Control, Beijing, China

**Keywords:** hypertension, transtheoretical model, blood pressure, medication adherence, clustered randomized controlled trial

## Abstract

**Background:**

Hypertension can result in great cardiovascular risk, while medication adherence and blood pressure control of patients were suboptimal. Therefore, we conducted a 12-month clustered randomized controlled trial to evaluate the effectiveness of transtheoretical model (TTM)-based health intervention mode on blood pressure of Chinese newly-diagnosed hypertensive patients.

**Methods:**

This trial was conducted in six primary healthcare centers, Shunyi District, Beijing, China from September 2016 to September 2017. A total of 400 patients were included and randomized, 194 patients in the control group and 206 patients in the intervention group. Patients in the intervention group received TTM-based health intervention and those in the control group received usual care. Multilevel modeling was used to adjust for clustering effect and repeated measurements.

**Results:**

Systolic blood pressure of patient was reduced by 4.534 mm Hg at 3-month follow-up [95% CI (−3.054, −1.403), *p* = 0.005], 3.982 mm Hg at 6-month follow-up [95% CI (−7.387, −0.577), *p* = 0.022], and 5.803 mm Hg at 12-month follow-up [95% (−9.716, −1.891), *p* = 0.004]. Diastolic blood pressure of patient was reduced by 3.383 mm Hg at 3-month follow-up [95% CI (−5.724, −1.042), *p* = 0.005], 0.330 mm Hg at 6-month follow-up [95% CI (−2.870, 2.210), *p* = 0.799], and 3.129 mm Hg at 12-month follow-up [95% CI (−6.048, −0.21), *p* = 0.036]. Medication adherence of patients was improved at all the three time points. For stages of change for taking medication, patients in the intervention group were 8.401-fold more likely to be in a higher stage at 3-month follow-up [95% CI (4.186, 16.862), *p* < 0.001]. The odds ratio of being in a higher stage was 8.454 at 6-month follow-up [95% CI (3.943, 18.123), *p* < 0.001] and 19.263 at 12-month follow-up [95% CI (7.979, 46.505), *p* < 0.001].

**Conclusion:**

Transtheoretical model-based health intervention might be a promising strategy to improve medication adherence of newly-diagnosed hypertensive patients in community.

## Introduction

Hypertension is a leading cause of morbidity and mortality in China. Uncontrolled blood pressure (BP) might cause substantial health consequences such as increased risks for stroke, heart diseases, and death (1–3). It also brings large economic burden on the whole society. In 2013, total health expenditure of China was 31,869 billion yuan, of which the direct economic burden of hypertension accounted for 6.6% (4). It was estimated that high systolic BP (SBP) accounted for 2.54 million deaths, which was the top risk factor for both the number of deaths and percentage of the disability-adjusted life years (DALYs) in 2017 in China (5). The National Center for Cardiovascular Diseases in China reported that 23.2% of the Chinese adults had hypertension according to the Chinese guideline, but only 15.3% hypertensive patients controlled their BP (6). With the high prevalence, poor control rate, and limited medical resources, it is very important to develop a suitable and efficient mode for primary medical staff to help them carry out hypertension management.

Transtheoretical model (TTM) proposed a dynamic and continuous changing process before completing a real behavior change, which could serve as a guidance to address different needs of patients at different stages of change. This model consists of four elements including stages of change, processes of change, decision balance, and self-efficacy. Stages of change classified readiness of an individual to change into five categories: precontemplation (PC) (having no intention to change for a certain behavior in the next 6 months), contemplation (having intention to change for a certain behavior in the next 6 months), preparation (preparing to change in the next 4 weeks), action (having adhered to a certain behavior, but for <6 months), and maintenance (indicating performing change more than 6 months) (7). Processes of change pointed out two strategies named cognition transition and behavior transition including ten methods. It explains how behavior change occurs. Whether an individual can transition from one stage to another stage, it depends on the processes of change of each stage. Cognition transition strategy consists of consciousness raising, dramatic relief, environmental reevaluation, self-reevaluation, social liberation and behavior transition strategy consists of helping relationships, self-liberation, counterconditioning, stimulus control, and reinforcement management. Decision balance means the judgment of the costs and benefits of adopting behavior change and self-efficacy refers to confidence of an individual in whether he can use his own skills to complete a certain behavior. Stages of change are the core of the model and the processes of change, decision-making balance, and self-efficacy are the components and the comprehensive intervention guiding principle.

Several studies have designed and implemented interventions based on TTM and obtained good effects such as successful weight loss among obesity adults and nutrition improve among mental disorder patients (8, 9). It also has been proved that TTM-based intervention could help hypertensive patients to improve their dietary and exercise (10, 11). But, there is rare study conducting intervention among hypertensive patients based on TTM focusing on medication taking, except a study performed in the United States in 2006 (12). It has been proved that various factors contributed to poor BP control, of which poor medication adherence was the most modifiable (13–15). Better medication adherence was directly linked to better BP control and decreased risks for cardiovascular diseases (16, 17). However, despite the importance of medication taking, a large number of hypertensive patients in China still had poor medication-taking behavior (18). Therefore, conducting an intervention from the perspective of improving the medication adherence of hypertensive patients may be the most efficient approach to obtain better BP control, especially for medical staff in community in China.

For newly-diagnosed hypertensive patients, medication taking is a new behavior to follow, so TTM-based intervention is suitable for them to complete the behavior transition from the beginning. Therefore, we conducted the clustered randomized controlled trial to: (1) evaluate the effectiveness of TTM-based intervention on BP control and (2) clear whether TTM-based intervention could promote the stages of change in taking medication.

## Materials and Methods

### Study Design and Participants

This study was approved by the Institutional Review Board of Peking University Health Science Center (IRB00001052-15040). Informed consent was obtained from all the participants before they were enrolled in this study.

A community-based clustered randomized controlled trial was conducted in six community healthcare centers in Shunyi District, Beijing, China from September 2016 to September 2017. We recruited patients with the help of the doctors in the community healthcare centers through the medical record information system. All the potential patients would be invited within 1 week to participate in this study. The six community healthcare centers were randomly assigned to the control group or the intervention group in a 1:1 ratio using concealed random allocation. Randomization was achieved with random numbers. Due to the nature of intervention, both the patients and healthcare providers were aware of their treatment allocation.

The sample size estimation was calculated by repeated measurement in PASS.11 software. The value of α was set as 0.05, power as 0.9, number of repeated measurements (M) as 4, and conditional correlation coefficient (ρ) as 0.8. The result showed that one group was expected to recruit 152 patients. Considering possibility of losses to follow-up, the sample size was expanded to 210 in each group.

Patients with essential hypertension who volunteered to participate, aged above 18 years, and newly diagnosed according to the codes 401-401.9 of the 9th version of International Classification of Diseases were included. Newly-diagnosed hypertensive patients were defined as those having a diagnosis of hypertension within half a year. We excluded those that failed or unable to sign informed consent, had mental disorders, and had secondary hypertension.

### Transtheoretical Model-Based Interventions and Usual Care

This intervention lasted 6 months. Three group sessions lasting 20–30 min each session were delivered to the intervention group every 3 months. The group session was conducted by the primary healthcare staff at each community healthcare center, with the help of a standardized PowerPoint slides. We assumed that patients at the beginning were at lower stages of medication adherence and as this study went on, a large number would transit to a higher stage. In specific, we assumed that patients in stage of PC and contemplation were in the majority at baseline. Three months after the first group session, patients were almost in the stage of preparation and action. Three months after the second group session, patients in the stage of action and maintenance were in the majority. The contents of group session were designed according to the assumed stages of change. The processes of change, decision-making balance, and self-efficacy were added into the content to promote the stages of change, as shown in Table 1. The staffs at each community healthcare center were called together to attend training sessions, which were conducted through standardized PowerPoint slides by the same person from the research group.

**Table 1:** Applying transtheoretical model (TTM) in design of the intervention.

**Time**	**Stages of change**	**Processes of change**	**Main contents of session**	**Objective**
Baseline	Pre-contemplation and contemplation	Consciousness raising Environmental reevaluation Dramatic relief Social liberation Self-reevaluation	a) Introduction of common knowledge and routine measures for blood pressure control, such as healthy lifestyle, medication. b) Consequences of uncontrolled blood pressure. c) How to correctly measure blood pressure.	a) Arise patients' awareness of taking medication. b) Let them recognize the benefits of medication adherence.
3-month	Preparation and action	Self-reevaluation Self-liberation Helping relationships Counter conditioning	a) Vivid examples s to address patients' wrong perceptions and psychological issues in terms of taking medication. b) Provided an electronic pill case to remind them taking medicine on time.	a) Decreasing perceived obstacles in TTM's decisional balance dimension. b) Enhance patients' intention and awareness of taking medication.
6-month	Action and maintenance	Reinforcement management Helping relationship Stimulus control	a) Vivid examples to help patients realize and correct their own potentially wrong medication adherences or perceptions. b) Helped patients to analyze the reasons of corresponding wrong behaviors, and help them to address the possible barriers. c) Encourage patients to share their own successful experiences.	a) Help maintain and avoid returning to the previous stage. b) Enhance patients' intention and awareness of taking medication.

Patients in the control group received usual care as required by the Chinese national basic public health service project (19). In this project, healthcare staff was required to provide at least four times of follow-up services to hypertensive patients including BP measurements and basic lifestyle and medication-taking instructions.

### Data Collection

Questionnaire surveys and physical examinations were performed at baseline, 3-month follow-up, 6-month follow-up, and 12-month follow-up. These surveys and examinations were conducted face-to-face either in the control group or in the intervention group. For the intervention group, the first three times of questionnaire surveys and physical examinations were conducted before the group session every time and patients were informed to participate in the fourth one for a special purpose. For the control group, patients just participate in the surveys and examinations in their community healthcare center every time.

#### Sociodemographic Characteristics

Sociodemographic and disease-related information were collected using a questionnaire including age, gender, marital status, family history, existence of comorbidity, and so on.

#### Stages of Change

Stages of change of patients for taking medication were measured with self-designed questionnaire. The questionnaire was based on TTM model and relevant literature (20). The only question assessed which stage patients were in. Five options for stages of change were provided. They were defined and scored as follows: 1 = PC (having no intention to take medication in the next 6 months); 2 = contemplation (having intention to take medication in the next 6 months); 3 = preparation (preparing to take medication in the next 4 weeks); 4 = action (having adhered to take medication, but for <6 months); and 5 = maintenance (insisting taking medication more than 6 months).

#### Blood Pressure

Blood pressure of patients was measured at each follow-up. BP was measured with Omron J710 automated BP machine during 7 am to 9 am at clinic visit of patient. Patients were told not to smoke and drink tea or coffee in 30 min before measurement. Consecutive seated BP with 2-min intervals was measured after patients resting for 15 min. The average of three values was taken.

### Statistical Analysis

Normality test was performed on continuous data. Those that followed the normal distribution were reported as mean ± SD and others were described by median and interquartile range. Categorical data were reported in frequency and proportion. The chi-squared test was used to determine whether characteristics differed significantly between the control group and the intervention group at baseline and to determine whether there is difference between patients insisting follow-up and those lost. The *t*-test was used to compare the difference in blood pressure between the control group and the intervention group at baseline, 3-month follow-up, 6-month follow-up, and 12-month follow-up.

Considering high attrition rate, multilevel modeling was adopted to deal with missing data and account for clustering and repeated measurements (21). Three-level modeling (measurements nested within individual nested within community healthcare centers) was applied to account for clustering and repeated measurements. In this model, a time dummy was created to indicate the time trend in the treatment and control groups and an interaction term between the intervention and time dummy was generated to represent the net effect of intervention impact on outcomes of patient. The analysis was adjusted for gender, age, education, income, and medical insurance. *p* < 0.05 was considered as statistically significant. Data were entered with EpiData 3.1 and statistically analysis was performed in Stata SE 14.1. The primary outcome is BP including systolic BP (SBP) and diastolic BP (DBP) and the secondary outcome is the stages of change for taking medication.

## Results

### Participation and Characteristics

**Figure 1:**
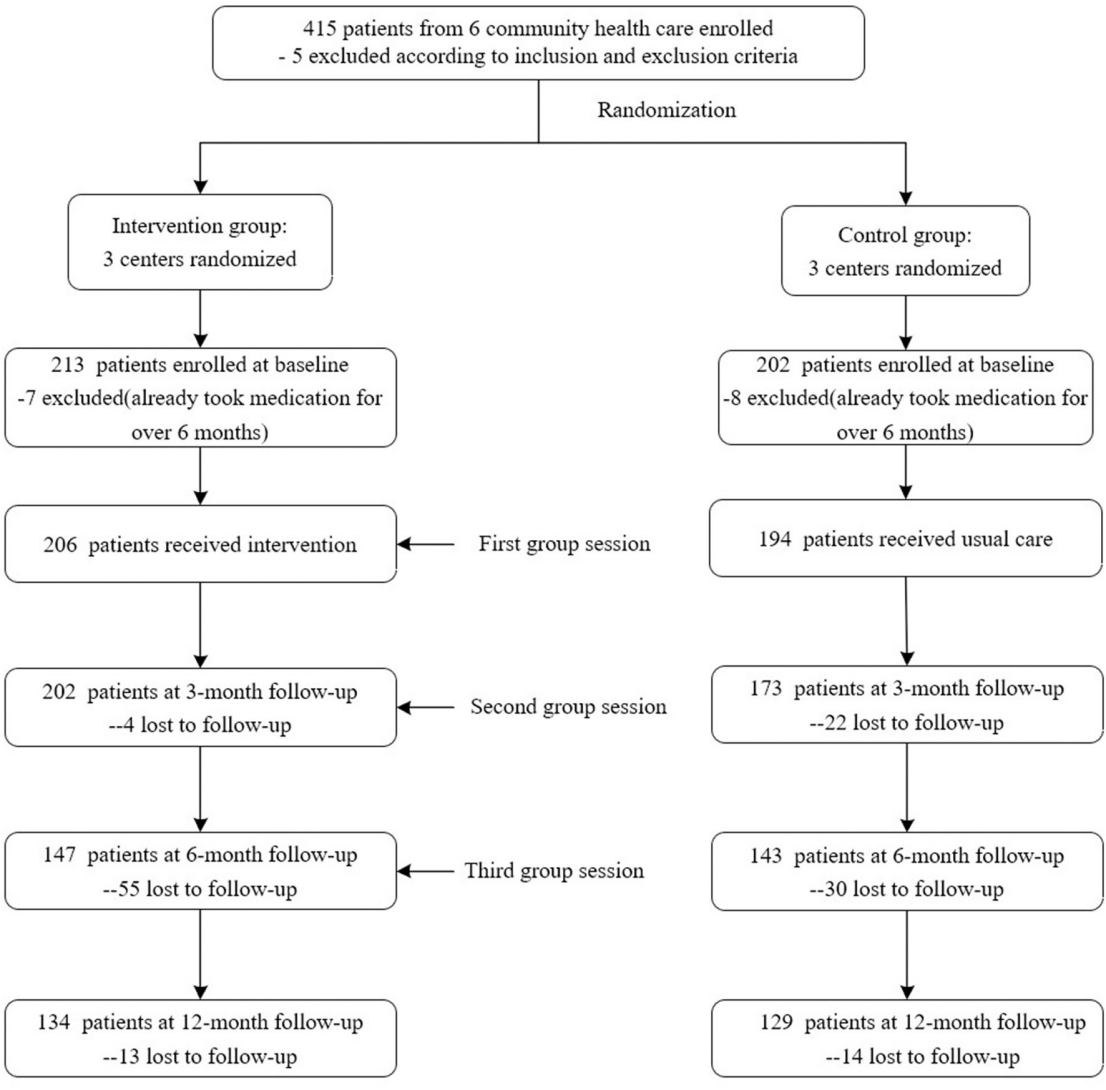
Participation flow chart.

A total of 420 patients were recruited, with 415 patients met the inclusion criteria. But, further validation based on the questionnaire showed that 15 patients have taken medication more than half a year, which means they have been diagnosed as hypertension more than half a year. So, these 15 patients should be excluded according to our criteria. Finally, 206 patients received the intervention and 194 received usual care. With follow-up, some participants failed to be tracked. The specific number was shown in Figure 1. The reasons of lost to follow-up included changes of contact information or home addresses and some patients also withdrew due to the relatively long study duration. We compared socioeconomic characteristics of patients between baseline and 3-month follow-up, baseline and 6-month follow-up, and baseline and 12-month follow-up, respectively. No significant difference was found in these time points.

At baseline, the mean age was 55.1 ± 8.5 years and female patients took a proportion of 70.2%. As shown in Table 2, patients in the control group and the intervention group did not differ significantly in terms of gender, age, education, marital status, existence of comorbidities, and family history. However, patients in the intervention group had better family income (*p* = 0.002) and insurance coverage (*p* < 0.001). DBP did not differ significantly at baseline for the two groups, but patients in the control group had significantly lower SBP (*p* = 0.005). In terms of stages of taking medication, there were substantially more patients in the control group (50%) compared with those in the intervention group (27.2%, *p* < 0.001) (Table 2).

**Table 2:** Baseline characteristics of participants.

**Characteristic**	**Control**	**Intervention**	***P***
	**(*N* = 194)**	**(*N* = 206)**	
**Gender**, ***N*** **(%)**			
Male	51 (26.3)	68 (33.0)	0.142
**Age**, ***N*** **(%)**			
<50	41 (21.1)	52 (25.2)	0.302
50–	83 (42.8)	94 (45.6)	
≥60	70 (36.1)	60 (29.1)	
**Education**, ***N*** **(%)**			
Primary school and below	34 (17.5)	22 (10.7)	0.118
Middle school	105 (54.1)	112 (54.3)	
High school	38 (19.6)	43 (20.9)	
University and above	17 (8.8)	29 (14.1)	
**Marital status**, ***N*** **(%)**			
Married	195 (94.7)	183 (94.3)	0.885
**Income monthly (RMB yuan)**, ***N*** **(%)**			
<1,500	39 (20.1)	21 (10.2)	**0.002**
1,500–	38 (19.6)	28 (13.6)	
3,000–	37 (19.0)	55 (26.7)	
5,000–	55 (28.4)	54 (26.2)	
≥10,000	25 (12.9)	48 (23.3)	
**Insurance**, ***N*** **(%)**			
New rural cooperative medical insurance	145 (74.7)	104 (50.5)	**<0.001**
Urban residents' basic medical insurance	17 (8.8)	28 (13.6)	
Urban employees' basic medical insurance	32 (16.5)	74 (35.9)	
**Existence of comorbidity**, ***N*** **(%)**			
Yes	107 (55.2)	125 (60.7)	0.263
**Family history**, ***N*** **(%)**			
Yes	129 (66.5)	141 (68.4)	0.677
SBP, Mean(SD)	137.2 (13.5)	141.1 (14.2)	**0.005**
DBP, Mean(SD)	85.0(10.4)	84.1(11.7)	0.474
**Stage of taking medication**, ***N*** **(%**)			
Pre-contemplation	53 (27.3)	83 (40.3)	<**0.001**
Contemplation	34 (17.5)	49 (23.8)	
Preparation	10 (5.2)	17 (8.3)	
Action	97 (50.0)	57 (27.7)	

*Bold values indicate P < 0.05*.

### Stages of Change in Intervention Group Behaved Better

**Figure 2:**
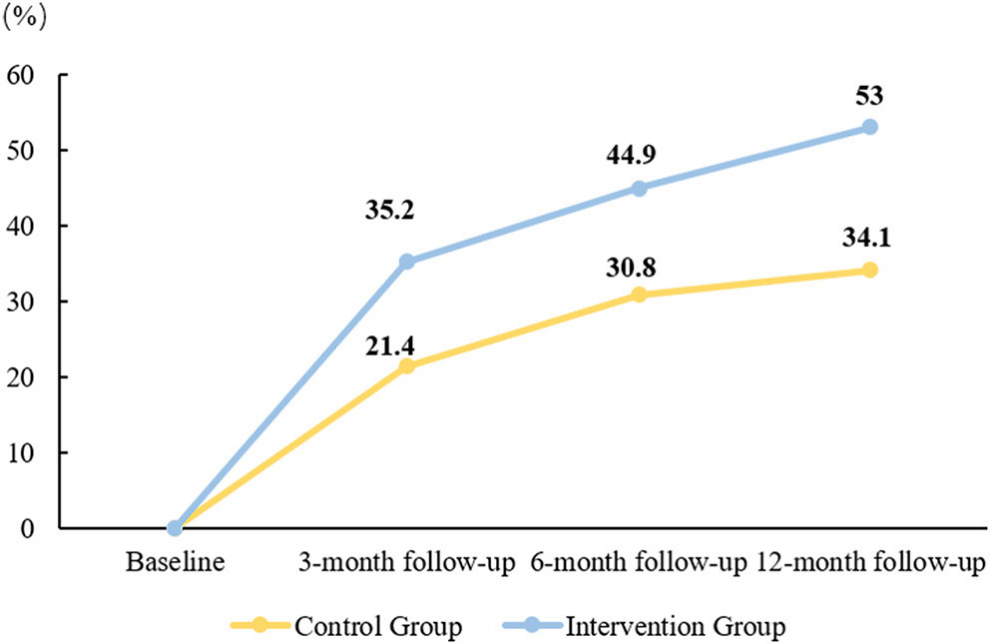
The proportion of patients in maintenance stage.

The proportion of patients in maintenance stage in the two groups showed an increasing trend and the increasing trend of the intervention group was better than that of the control group as a whole (Figure 2).

**Figure 3:**
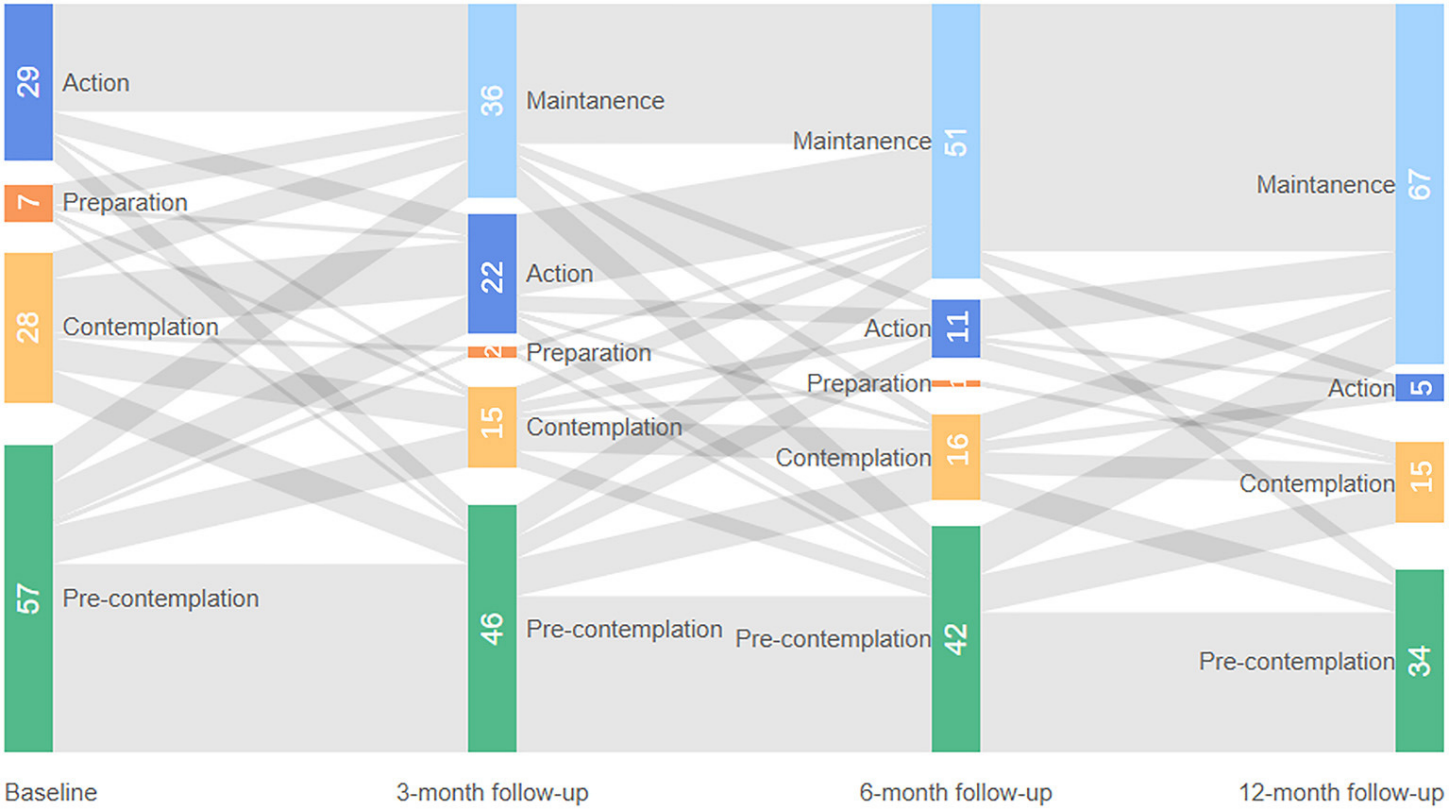
Stage of change for medication taking at all time points in the intervention group.

**Figure 4:**
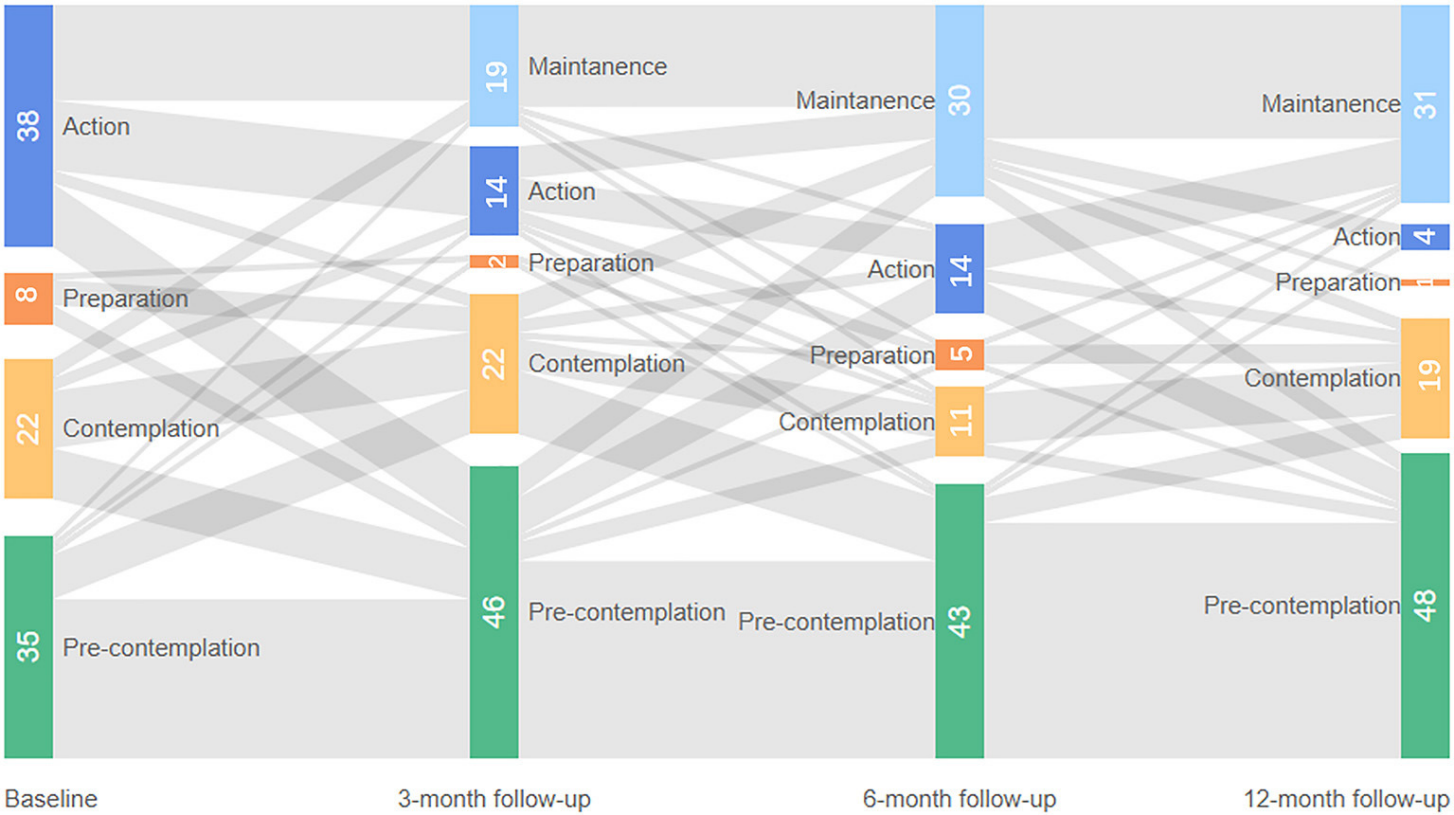
Stage of change for medication taking at all time points in the control group.

Stages of change for taking medication at baseline, 3-month follow-up, 6-month follow-up, and 12-month follow-up were given in Figures 3, 4. These two figures illustrated the patients who completed all the follow-up: 121 patients in the intervention group and 103 patients in the control group. As shown in figures, the shaded part represents the flow of people into different stages of change. Generally, more patients in the intervention group moved forward in terms of medication taking at three time points and more patients in the control group moved backward from TTM stage compared with their last measurement. As the proportion of patients in the contemplation stage and preparation stage was relatively small, these two groups of patients were merged in the multilevel analysis. Patients in the action and maintenance stages were also merged due to the same reason. Therefore, we had three categories of medication adherence in the final analysis: PC, contemplation/preparation (CP), and action/maintenance (AM).

### Transtheoretical Model-Based Intervention Reduced BP Effectively

Results showed that SBP of patients in the intervention group was statistically higher than those in the control group at baseline (*p* < 0.05), but there was insignificant difference at 12-month follow-up (*p* = 0.545). DBP of patients in the intervention group was significantly lower than those in the control group at 3-month follow-up, 6-month follow-up, and 12-month follow-up. SBP of patients in the two groups decreased first and then increased and showed a downward trend as a whole. SBP of the intervention group decreased by 8.0 mm Hg and the control group decreased by 0.5 mm Hg; in terms of DBP, the intervention group decreased by 5.8 mm Hg totally, while the control group showed a trend of first rising, then falling, and then rising. On the whole, it showed the decrease of 1.8 mm Hg in the control group, as seen in Table 3.

**Table 3:** Systolic blood pressure and diastolic blood pressure.

**Blood pressure**	**Group**	**Baseline**	**3-month follow-up**	**6-month follow-up**	**12-month follow-up**
Systolic	Control	137.2 ± 13.5	136.7 ± 13.3	133.4 ± 14.0	136.7 ± 15.0
	Intervention	141.1 ± 14.2	135.2 ± 13.7	131.6 ± 14.9	133.1 ± 15.1
	t	−2.802	1.083	1.0301	1.929
	*P*	0.005	0.280	0.304	0.545
Diastolic	Control	85.0 ± 10.4	86.1 ± 8.2	80.6 ± 10.8	83.2 ± 9.1
	Intervention	84.2 ± 11.7	81.2 ± 8.9	78.2 ± 9.6	78.4 ± 9.2
	t	0.717	5.518	1.987	4.2801
	*P*	0.474	<0.001	0.048	<0.001

### Multilevel Model Showed Effective TTM-Based Intervention

Results of multilevel modeling indicated that after adjusting for gender, age, education, income, and medical insurance, stages of change of patients for taking medication were significantly improved for the intervention group at all the three time points. At 3-month follow-up, patients in the intervention group were 8.401-fold more likely to be in a higher stage [PC vs. CP or CP vs. AM, 95% CI (4.186, 16.862), *p* < 0.001]. The odds ratio of being in a higher stage was 8.454 [95% CI (3.943, 18.123), *p* < 0.001] at 6-month follow-up and 19.263 at 12-month follow-up [95% CI (7.979, 46.505), *p* < 0.001] (Table 4). SBP of patient was reduced by 4.534 mm Hg at 3-month follow-up [95% CI (−3.054, −1.403), *p* = 0.005], 3.982 mm Hg at 6-month follow-up [95% CI (−7.387, −0.577), *p* = 0.022], and 5.803 mm Hg at 12-month follow-up [95% (−9.716, −1.891), *p* = 0.004]. The intervention effect for DBP was significant at 3-month follow-up and 12-month follow-up, but not for 6-month follow-up. This intervention reduced DBP of patients by 3.383 mm Hg at 3-month follow-up [95% CI (−5.724, −1.042), *p* = 0.005] and 3.129 mm Hg at 12-month follow-up [95% CI (−6.048, −0.21), *p* = 0.036]. The intervention effect at 6-month follow-up was −0.330 mm Hg, but it was not statistically important. Compared with patients in the PC stage, those at AM stage had a significantly lower DBP [*B* = −1.488, 95% CI (−2.826, −0.149), *p* = 0.029] (Table 4).

**Table 4:** Effects of TTM-based intervention on stage of change, systolic blood pressure, and diastolic blood pressure.

**Variable**	**Stages of change**			**SBP**			**DBP**		
	**OR**	**95%CI**	***P***	**β**	**95%CI**	***P***	**β**	**95%CI**	***P***
**Time points**
3-month	0.499	[0.302, 0.826]	0.007	−0.802	[−3.054, 1.451]	0.486	1.031	[−0.654, 2.715]	0.230
6-month	0.573	[0.336, 0.979]	0.042	−4.158	[−6.567, −1.748]	0.001	−4.580	[−6.567, −2.782]	<0.001
12-month	0.393	[0.209, 0.739]	0.004	−0.418	[−3.285, 2.45]	0.775	−1.858	[−3.997, 0.281]	0.089
**Group**
Intervention	0.255	[0.029, 2.242]	0.218	3.373	[−0.85, 7.596]	0.117	−2.105	[−5.685, 1.474]	0.249
Time*group interaction
3-month	8.401	[4.186, 16.862]	<0.001	−4.534	[−3.054, −1.403]	0.005	−3.383	[−5.724, −1.042]	0.005
6-month	8.454	[3.943, 18.123]	<0.001	−3.982	[−7.387, −0.577]	0.022	−0.330	[−2.87, 2.21]	0.799
12-month	19.263	[7.979, 46.505]	<0.001	−5.803	[−9.716, −1.891]	0.004	−3.129	[−6.048, −0.21]	0.036
**Stage of change**
Contemplation/preparation				0.927	[−1.115, 2.97]	0.374	1.488	[0.018, 2.958]	0.047
Action/maintenance				−2.000	[−3.896, −0.103]	0.039	−1.488	[−2.826, −0.149]	0.029

## Discussion

Our main objective in this study was to evaluate the effectiveness of TTM-based intervention on compliance of taking medication and BP control in newly-diagnosed hypertensive patients. This TTM-based intervention could help medical staff in community to promote the newly-diagnosed hypertensive patients to take medication and better control their BP. But, there was a relatively high dropout rate, so the real scope is relatively limited compared with the scope designed in the beginning. This study focuses on newly-diagnosed hypertensive patients, so it should be cautious when extrapolating the conclusion.

In terms of our primary outcome, we significantly improved SBP of patients by 4.534 mm Hg at 3-month follow-up, 3.982 mm Hg at 6-month follow-up, and 5.803 mm Hg at 12-month follow-up. Furthermore, DBP of patients was also significantly improved at 3-month follow-up (3.383 mm Hg) and 12-month follow-up (3.129 mm Hg). Therefore, this study results indicated that TTM-based health intervention could substantially improve BP level of hypertensive patients by promoting their taking medication. Although our intervention only lasts for 6 months, BP level of hypertensive patients and status of taking medication continued to improve 6 months after intervention and the extent of improvement reached the maximum at 12-month follow-up. This result suggested that TTM-based health intervention was effective both in a short term and in a long term.

The short-term effects of TTM-based various interventions were examined in different ethnicities with different diseases (12, 22, 23). However, to the best of our knowledge, the evidence in terms of a Chinese hypertensive population was scarce. A Malaysian TTM-based lifestyle intervention decreased SBP of patients by 5.6 mm Hg [95% CI (5.8, 9.4), *p* < 0.001] and DBP of patients by 6.1 mm Hg [95% CI (3.9, 6.5), *p* < 0.001] in a 6-month intervention (22). Although this study focused on lifestyles of patients such as physical activities, salt, and fruits and vegetable intakes rather than taking medication, it could still validate the short-term effectiveness of TTM-based interventions. Furthermore, another TTM-based behavioral intervention (diet/exercise/medication taking) conducted among American hypertensive veterans improved SBP of patients by approximately 4.7 mm Hg in 6 months of intervention, which was similar to SBP decrease in this study (23). Moreover, Johnson et al. also found that a 6-month TTM-based intervention could improve English nonadherent medication-taking stages of hypertensive patients (12). Compared with those studies, this study further confirmed that TTM-based interventions were effective in a short term such as in 3 and 6 months and we also add to the existing evidence that TTM-based interventions could continue to have an impact even if the intervention stopped.

Apart from this study duration, this study was distinct in terms of the patient population examined. First of all, compared with the other two studies evaluating the effectiveness of TTM-based intervention on BP control of patients, most subjects in this study were less-educated rural females, while their participants were mostly well-educated males and one only included veterans (22, 23). Therefore, results from this study could well complement the research gap in terms of the effectiveness of TTM-based interventions on less-educated females. Second, compared with other studies that tended to educate nonadherent patients, we focused on the newly-diagnosed hypertensive patients. The importance of antihypertensive in disease management of patients was well established and in most cases, newly-diagnosed hypertensive patients have not developed a medication-taking habit. Therefore, helping them to form a good medication adherence at the starting point of the disease journey could benefit patients to a great extent. In this study, compared with patients in the control group that only received usual care, those in the intervention group were 19-fold more likely to be in a higher stage of taking medication. To conclude, TTM-based intervention could substantially improve newly-diagnosed medication adherence of patients.

This study also had some limitations. First of all, the attrition rate was relatively high, although we tried to use multilevel modeling to overcome this issue. The attrition rate in this study was slightly lower than the TTM-based lipid-lowering and antihypertensive medication adherence studies conducted by Johnson et al. (12, 24). Second, stages of change of patients for taking medication were self-reported, therefore there might exist some bias. Third, the factors of lifestyle including diet, exercise, smoking, and drinking were not controlled in this study. Last, the baseline differences and imbalance between patients in the control group and the intervention group could also affect the results.

## Conclusion

Transtheoretical model-based intervention could significantly improve the stage of change for taking medication and BP control in newly-diagnosed Chinese hypertensive patients. This study suggests that in primary care settings, appropriate interventions such as TTM-based intervention should be provided to newly-diagnosed hypertensive patients and make them to realize the importance of antihypertensive before they form medication-taking habits.

## Data Availability Statement

The raw data supporting the conclusions of this article will be made available by the authors, without undue reservation.

## Author Contributions

PC, YS, and XYS: contributed to the conception and design of this study. PC, YS, and CH: contributed to the acquisition, analysis, or interpretation of data. PC and YS: drafted the manuscript and they are co-first authors. CH and XYS: critically revised the manuscript. All authors gave final approval and agreed to be accountable for all the aspects of this study ensuring integrity and accuracy.

## Funding

This study was funded by the Natural Science Foundation of Beijing (Grant No. 7202087).

## Conflict of Interest

The authors declare that the research was conducted in the absence of any commercial or financial relationships that could be construed as a potential conflict of interest.

## Publisher's Note

All claims expressed in this article are solely those of the authors and do not necessarily represent those of their affiliated organizations, or those of the publisher, the editors and the reviewers. Any product that may be evaluated in this article, or claim that may be made by its manufacturer, is not guaranteed or endorsed by the publisher.
